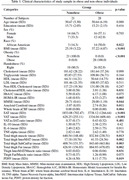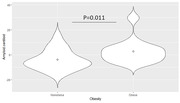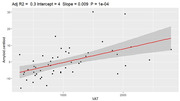# The Association between the Body Fat Localization, Insulin Resistance, and Amyloid Burden in Midlife

**DOI:** 10.1002/alz.094065

**Published:** 2025-01-09

**Authors:** Mahsa Dolatshahi, Paul K. Commean, Mahshid Naghashzadeh, Caitlyn Nguyen, LaKisha Lloyd, Sara Hosseinzadeh Kasani, Abby McBee‐Kemper, Jake Weeks, Claude Sirlin, Lael Ceriani, Xu Yifei, Bettina Mittendorfer, Jingxia Liu, Tammie L.S. Benzinger, Joseph E. Ippolito, John C. Morris, Cyrus A. Raji

**Affiliations:** ^1^ Mallinckrodt Institute of Radiology, Washington University in St. Louis, St. Louis, MO USA; ^2^ Washington University in St. Louis, St. Louis, MO USA; ^3^ University of California, San Diego, La Jolla, CA USA; ^4^ Washington University in Saint Louis, Saint Louis, MO USA; ^5^ Missouri University School of Medicine, Columbia, MO USA; ^6^ Washington University in St. Louis School of Medicine, St. Louis, MO USA; ^7^ Knight Alzheimer Disease Research Center, St. Louis, MO USA

## Abstract

**Background:**

Obesity in midlife is a risk factor for developing Alzheimer disease later in life. However, the metabolic and inflammatory effects of body fat varies based on its anatomical localization. In this study, we aimed to investigate the association of MRI‐derived abdominal visceral and subcutaneous adipose tissue (VAT and SAT), liver proton‐density fat fraction (PDFF), thigh fat‐to‐muscle ratio (FMR), and insulin resistance with whole‐brain amyloid burden in cognitively normal midlife individuals.

**Method:**

A total of 49 cognitively normal midlife individuals (Age: 50.65±5.77 years, 61.2% female, BMI: 32.0±67.34, 57.1% obese) underwent brain PET scan, body MRI, and metabolic assessment. Homeostatic Model Assessment for Insulin Resistance (HOMAIR) was used for measuring insulin resistance. Dynamic amyloid imaging was performed with a bolus injection of ∼15mCi [11C]PiB, followed by a 60‐min scan. Data from the 30‐60 minute post‐injection window was used for calculating whole‐brain amyloid centiloid. VAT and SAT were semi‐automatically segmented using an in‐house MATLAB‐based software. A trained U‐Net convolutional neural network (CNN) model was used to calculate the hepatic PDFF maps from abdominal T1‐weighted images. After preprocessing and N4ITK bias correction on mid‐thigh slices between the ischial ramus and the medial knee condyle, a MATLAB program was used for segmenting thigh total fat including subcutaneous, inter‐, and intra‐muscular fat, and muscle volumes. Total thigh fat‐to‐muscle ratio (FMR) was calculated. Using Spearman correlation test, the association between whole‐brain amyloid centiloid and BMI, HOMAIR, VAT, SAT, PDFF, and FMR was assessed, with age and sex as covariates.

**Result:**

Obese individuals had a higher amyloid burden compared to the non‐obese (p = 0.011). Whole‐brain amyloid centiloid values were significantly associated with VAT (rho = 0.62, p<0.0001) and HOMAIR (rho = 0.56, p = 0.013), but not other fat metrics. A mediation analysis showed significant direct effect of VAT (p = 0.02) on amyloid burden, while the indirect VAT effects mediated by HOMAIR were non‐significant (p = 0.28).

**Conclusion:**

Obesity, higher visceral fat and insulin resistance, but not BMI, subcutaneous abdominal fat, liver fat, or thigh fat, are associated with higher whole‐brain amyloid burden in midlife. This highlights the importance of anatomical characterization of body fat for Alzheimer disease risk, where visceral fat shows a strong relationship with amyloid pathology.